# Kirschner wire versus external fixation in the treatment of proximal humeral fractures in older children and adolescents: a comparative study

**DOI:** 10.1186/s12891-023-07037-x

**Published:** 2023-11-18

**Authors:** Yu Wang, Qian Wang, Wuyi Yao, Jingxin Zhao, Xiaobin Zhao, Man He

**Affiliations:** 1 Department of Orthopedics, Affiliated Hospital of Chengde Medical University, Chengde, Hebei 067000 PR China; 2 Department of Radiology, Affiliated Hospital of Chengde Medical University, 36 Nanyingzi Street, Chengde, Shuangqiao District, Hebei 067000 PR China; 3 Department of Rehabilitation, Affiliated Hospital of Chengde Medical University, Chengde, Hebei 067000 PR China

**Keywords:** Proximal humeral fracture, Older children, Adolescent, Kirschner wire, External fixation

## Abstract

**Objective:**

The purpose of this study was to compare the therapeutic effects of Kirschner wire fixation and external fixation in the treatment of proximal humeral fractures in older children and adolescents.

**Methods:**

A retrospective analysis was performed on the clinical data of older children and adolescents who underwent surgery at our institution for proximal humeral fractures between April 2014 and May 2022. One group (n = 28) underwent fracture reduction and Kirschner wire fixation, and the other group (n = 23) underwent external fixation. During the follow-up, the differences in shoulder joint function between the two groups were compared by analysing Quick Disabilities of the Arm, Shoulder, and Hand (Quick DASH) and Constant-Murley scores. Postoperative complications were also recorded.

**Results:**

The operation time of the Kirschner wire group was shorter than that of the external fixation group (69.07 ± 11.34 min vs. 77.39 ± 15.74 min, *P* = 0.33). The time to remove the fixator in the external fixation group was shorter than that in the Kirschner wire group (6.74 ± 1.57 vs. 7.61 ± 1.22, *P* = 0.032). The Quick DASH score and Constant-Murley score of the patients in the external fixation group were significantly better than those in the Kirschner wire group at 3 months after surgery (5.63 ± 4.33 vs. 8.93 ± 6.40, *P* = 0.040; 93.78 ± 2.43 vs. 91.75 ± 2.15, *P* = 0.003). There was no significant difference in the Quick DASH score or Constant-Murley score between the patients in the external fixator group and those in the Kirschner wire group at 9 months after the operation (2.77 ± 3.14 vs. 3.17 ± 3.68, *P* = 0.683; 97.39 ± 1.80 vs. 96.57 ± 2.15, *P* = 0.152). The most common complication of the two groups was pin tract infection. The incidence rate of infection was higher in the external fixation group than that in the Kirschner wire group (9 vs. 4, *P* = 0.043).

**Conclusion:**

Both Kirschner wire fixation and external fixation of N-H III and IV proximal humeral fractures in older children and adolescents produce good outcomes. External fixation is a preferred surgical treatment option for paediatric proximal humerus fractures because early mobilization of the affected limb can be realized.

**Supplementary Information:**

The online version contains supplementary material available at 10.1186/s12891-023-07037-x.

## Introduction

Paediatric proximal humeral fracture accounts for approximately 2% of all paediatric fractures, and the incidence rate is lower than that of supracondylar humerus fracture, ulnar and radial fracture, and distal radial fracture in children, with an average annual incidence of 31.4/100,000 [1]. Proximal humerus fractures most commonly occur between 11 and 15 years of age [2]. Age plays an important role in considering the treatment of proximal humerus fractures in children [3]. In young children with proximal humeral fractures, most of them will experience satisfactory results after conservative treatment because the proximal humerus has greater growth potential and plasticity [4]. The treatment of proximal humeral fractures with significant displacement in older children and adolescents is relatively complex, and the optimal treatment method remains a topic of discussion [5]. With increasing patient age, the plasticity of bones gradually decreases [1]. For children with large fracture displacement, surgery is needed to restore fracture alignment to restore normal joint function [6]. For this kind of patient, the healing time of the fracture also increases correspondingly, requiring a longer immobilization time [7]. At the same time, it is difficult to maintain the fracture position with nonoperative treatment, and limb immobilization causes unbearable psychological discomfort in patients, which is also one of the reasons why many authors have recommended surgical treatment of proximal humeral fractures in older children and adolescents in recent years.

The operation rate for paediatric proximal humeral fracture has increased significantly in the past decade. A recent study showed that the operation rate for paediatric proximal humeral fractures in general hospitals is 43.2%, while the rate of surgery in specialized hospitals that treat paediatric fractures alone is 11.9% [8]. Surgical treatment for fractures of the proximal humerus in children has been reported to have relatively satisfactory results [1, 9].

Surgical treatment for paediatric proximal humerus fractures include fixation with percutaneous Kirschner wire, elastic intramedullary nail, screw, and locking plate. Among them, percutaneous Kirschner wire fixation after fracture reduction is the most common surgical method [10]. In recent years, external fixation has been performed to treat paediatric proximal humeral fracture in some studies, which has become another optional surgical method.

As the only teaching hospital in the region that specializes in treating children’s orthopaedic diseases, we initially performed percutaneous Kirschner wire fixation as the surgical method for treating paediatric proximal humeral fracture. In 2016, we began to use external fixation to treat displaced proximal humeral fractures in older children and adolescents. The purpose of this study was to compare the therapeutic effect of Kirschner wire fixation and external fixation in the treatment of proximal humeral fractures in older children and adolescents and to summarize the precautions in the surgical treatment of paediatric proximal humeral fracture.

## Methods

### Patients

The clinical data of 51 children and adolescents with proximal humeral fractures who were treated at the Affiliated Hospital of Chengde Medical University from April 2014 to May 2022 were retrospectively analysed. The inclusion criteria for this study were age between 10 and 15 years and Neer-Horowitz (N-H)III and IV proximal humeral fractures. The N-H classification divides children’s proximal humeral fractures into four types: Type I (displacement of the fractures is less than 5 mm; Type II (fracture displacement is greater than 5 mm but less than 1/3 of the width of the humeral shaft); Type III (fracture displacement is nearly 2/3 of the width of the humeral shaft); and Type IV (fracture displacement exceeds 2/3 of the width of the humeral shaft). Two distinguished variants of proximal humerus fractures were included: epiphyseal injury and metaphyseal fracture. Pathological fracture and open fracture were excluded.

Twenty-eight patients were treated with K-wires, and 23 patients were treated with external fixation. The demographic and clinical characteristics of the patients are summarized in Table 1. Ethics approval and consent to participate - The study has been performed in accordance with the Declaration of Helsinki. Informed consent was obtained from all subjects and/or their legal guardian(s) in case of minors (below 16 years of age). The present study was approved by the Ethics Committee of Affiliated Hospital of Chengde Medical University. (Ethical approval NO. CYFYLL2022506).

**Table 1:** The demographic data of the two groups

Indicator	Kirschner wire	External fixation	t/x^2^	*P*
Number(n)	28	23		
Sex(male : female)*	18: 10	16:7	0.158	0.691
Years	11.96 ± 1.50	12.39 ± 1.47	0.027	0.313
Side(left : right)*	17:11	15:8	0.110	0.741
BMI	21.38 ± 4.11	22.00 ± 5.77	1.521	0.654
Time between injury and operation(day)	2.80 ± 3.39	1.99 ± 1.25	2.825	0.280
Hospitalization time (day)	7.07 ± 3.70	7.30 ± 4.96	0.434	0.849
Epiphyseal injury/metaphyseal fracture*	19:9	15:8	0.004	0.842
Operation time(min)	69.07 ± 11.34	77.39 ± 15.74	2.191	0.033
Times of fluoroscopy(n)	30.82 ± 9.05	33.91 ± 9.22	1.204	0.234
Open reduction ratio(%)	9(32.1%)	6(26.1%)	0.223	0.637
Amount of blood loss(ml)	29.93 ± 5.74	27.30 ± 3.91	1.864	0.068
Neer Horowitz(N-H)type III:IV*	10:18	14:9	3.21	0.073
Time to remove fixator after the operation(week)	7.61 ± 1.22	6.74 ± 1.57	2.214	0.032

*Use the chi-square test to calculate the *P* value

A *P* value < 0.05 was statistically significant

### Surgical techniques

The patients were in the supine position. After general anaesthesia, the elbow joint was flexed, the upper arm was pulled longitudinally in an internal rotational and adduction position, and the humerus shaft was simultaneously pushed back. Satisfactory reduction was confirmed by C-arm fluoroscopy. If closed reduction was difficult (3 attempts), open reduction was performed.

**Fig. 1:**
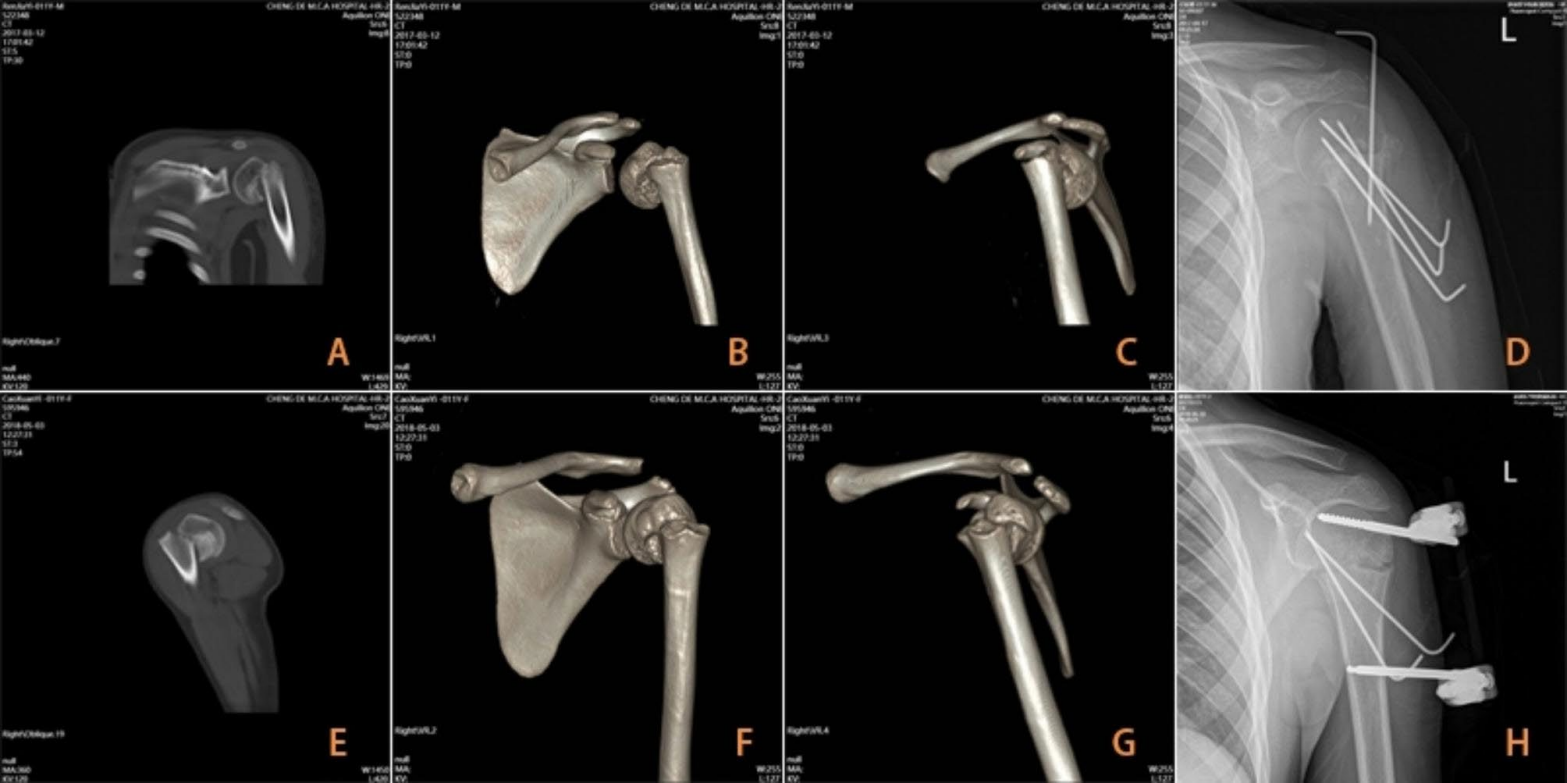
(**A**) Preoperative 2D-CT of a 11-year-old male patient with N-H IV proximal humeral fracture. (**B**) and (**C**) Preoperative 3D-CT of proximal humerus fracture. (**D**) Postoperative X-ray of proximal humerus fracture with Kirschner wires. (**E**) Preoperative 2D-CT of a 11-year-old female patient with N-H IV proximal humeral fracture. (**F**) and (**G**) Preoperative 3D-CT of proximal humerus fracture. (**H**) Postoperative X-ray of proximal humerus fracture with the external fixation

In the Kirschner wire group, two or three 1.8 or 2.0 mm Kirschner wires were used to drill into the humeral head from the humeral shaft in the reverse direction. Ideally, one Kirschner wire was inserted from the outside of the tendon of the long head of the biceps brachii muscle and fixed from the front to the rear. The other 1–2 wires were inserted close to the lateral side and fixed from the outside-in direction (Fig. S1). Depending on the situation, a Kirschner wire can also be inserted from the humeral head to the humeral shaft. After satisfactory fixation, satisfactory fracture reduction and the position of the Kirschner wire was confirmed by repeat C-arm fluoroscopy. After confirmation, the Kirschner wire was cut and bent to stay outside the skin. A U-shaped plaster of the upper arm was used to fix the affected shoulder joint (Fig. 1A-D).

The surgical technique described in our previous article [5] was used in the external fixation group. The procedure was as follows: we inserted a 4.0 mm cancellous bone Schanz screw in the humeral head after satisfactory reduction of the fracture. C-arm fluoroscopy was used to ensure the proper position and depth of the Schanz screw. The proximal Schanz screw was inserted through a mini-incision. Another incision of 2–3 cm was made at the distal end of the fracture, and the soft tissue was separated from the bone surface. A 4.0 mm diameter self-drilling Schanz screw was directly inserted into the distal part of the fracture using an electric drill. The external fixator was held in place by a connecting rod after fluoroscopic fracture reduction and a satisfactorily placed pin. One or two 1.8 or 2.0 mm anti-rotating Kirschner wires were then inserted. Anti-rotating Kirschner wires were fixed together with the external fixator using clips (Fig. S2). After surgery, the shoulder joint was immobilized using a sling, and after the discomfort was reduced, the patient was allowed to resume mild activity involving the shoulder joint (Fig. 1E-H).

### Postoperative treatment, re-examination, and evaluation

Patients were followed up in outpatient clinics in the second week, fourth week, second month, third month, and ninth month after surgery and then followed up irregularly. During the follow-up, healing of the fracture was observed by X-ray or CT, and the incidence of complications was also observed. The patients independently performed shoulder joint function training exercises on their own terms with the assistance of their guardians. Surgeons engaged in timely communication and guided patients in performing functional exercise through mobile phones and WeChat. After fracture healing, the external fixators were removed in the outpatient clinic without anaesthesia. Shoulder function was comprehensively evaluated, and the Quick DASH score and the Constant-Murley score of the shoulder were determined 3 months and 9 months after the operation. These two scores are suitable for the functional evaluation of older children and adolescents after upper limb surgery [11].

### Statistical analysis

The data were analysed by SPSS 22.0 software. For each of the analysed variables, descriptive statistics, including means and frequencies, were computed. The independent-sample t test for methodological data or the chi-square test for counting data, as applicable, were used to evaluate the treatment results between the two groups. A *P* value < 0.05 was considered statistically significant.

## Results

The 51 patients were followed up for an average time of 18.92 ± 3.27 months. The average age of the patients in the Kirschner wire group was 11.96 ± 1.50 years old, and the average age of the patients in the external fixation group was 12.39 ± 1.47 years old. There were no significant differences between the two groups in terms of sex, age, affected side, BMI, time from injury to operation, length of hospital stay, ratio of epiphyseal injury/metaphyseal fracture, operation time, number of fluoroscopy procedures, open reduction ratio, amount of blood loss or N-H classification, as shown in Table 1.

The operation time of the Kirschner wire group was shorter than that of the external fixation group (69.07 ± 11.34 min vs. 77.39 ± 15.74 min, *P* = 0.33). The fractures in both groups healed smoothly, and the time for removing the fixator in the external fixation group was shorter than that in the Kirschner wire group (6.74 ± 1.57 weeks vs. 7.61 ± 1.22 weeks, *P* = 0.032) (Table 1). The Quick DASH score and Constant-Murley score of the patients in the external fixation group were significantly better than those of the patients in the Kirschner wire group at 3 months after surgery (5.63 ± 4.33 vs. 8.93 ± 6.40, *P* = 0.040; 93.78 ± 2.43 vs. 91.75 ± 2.15, *P* = 0.003). At 9 months after the operation, there was no significant difference in the Quick DASH score or the Constant-Murley score between the patients in the external fixation group and those in the patients in the Kirschner wire group (2.77 ± 3.14 vs. 3.17 ± 3.68, *P* = 0.683; 97.39 ± 1.80 vs. 96.57 ± 2.15, *P* = 0.152). The function of the patients’ shoulder joints steadily improved with the lengthening of the postoperative rehabilitation period. The Kirschner wire group’s Quick DASH score at 9 months after surgery was considerably better than that at 3 months after surgery (3.17 ± 3.68 vs. 8.93 ± 6.40, *P* < 0.001). The Quick DASH score of the external fixation group at 9 months after surgery was noticeably better than that at 3 months after surgery (2.77 ± 3.14 vs. 5.63 ± 4.33, *P* = 0.014, Table 2). In terms of complications, the most common complication of the two groups was pin tract infection. The incidence rate of pin tract infection in the external fixation group was higher than that in the Kirschner wire group (9 vs. 4, *P* = 0.043, Table 3). It was cured by local dressing changes and later removal of the fixator. In the Kirschner wire group, there were two patients with slight displacement of the Kirschner wire, which did not affect fracture healing or cause other adverse effects. There was no other infection, secondary displacement of the fracture, vascular or nerve injury, fracture nonunion, joint stiffness, osteonecrosis or other complications. Both groups of patients returned to their preinjury living status without residual shoulder deformity or pain.

**Table 2:** Shoulder joint function between the two groups

Indicator	Kirschner wire	External fixation	*P* within groups
Quick DASH score of 3 months after surgery	8.93 ± 6.40	5.63 ± 4.33	0.040
Quick DASH score of 9 months after surgery	3.17 ± 3.68	2.77 ± 3.14	0.683
***P*** between groups	<0.001	0.014	
Constant score of 3 months after surgery	91.75 ± 2.15	93.78 ± 2.43	0.003
Constant score of 9 months after surgery	96.57 ± 2.15	97.39 ± 1.80	0.152
***P*** between groups	<0.001	<0.001	

A *P* value < 0.05 was statistically significant

**Table 3:** Complications in two groups

Complication	Kirschner wire (n = 28)	External fixation(n = 23)	*P*
Early complications			
Pin tract infection	4(14.3%)	9(39.1%)	0.043
Other infection	0	0	>0.999
Displacement of the fixation	2(7.1%)	0(0%)	0.495
Secondary displacement of the fracture	0	0	>0.999
Vascular and nerve injury	0	0	>0.999
Late complications			
Fracture nonunion	0	0	>0.999
Joint stiffness	0	0	>0.999
Osteonecrosis	0	0	>0.999

A *P* value < 0.05 was statistically significant

## Discussion

Proximal humeral fractures in children include proximal humeral epiphyseal injuries and metaphyseal fractures. A recent systematic review and meta-analysis of paediatric proximal humerus showed that the incidence of proximal humeral epiphyseal injuries was approximately twice as high as that of metaphyseal fractures [11]. Proximal humeral epiphyseal injuries account for 3 − 6.7% of all epiphyseal injuries, and the incidence rate is approximately 2.2–4.5/1000 cases of epiphyseal injuries every year [6]. The Salter-Harris classification of epiphyseal injury is usually used to classify proximal humeral epiphyseal injury in children. Most children and adolescents over 11 years old with proximal humeral epiphyseal injuries have Salter-Harris II injuries, and few have III- and IV-type fractures [1, 12]. The shoulder joint capsule of children attaches along the lateral side of the proximal epiphyseal plate of the humerus and then attaches downwards across the epiphyseal plate to the medial side of the metaphysis. This anatomical structure explains the high incidence of Salter-Harris II epiphyseal separation fractures, which contain a wedge-shaped Thurston Holland fragment in the metaphysis. In our study, the number of cases of epiphyseal injuries was also higher than that of metaphyseal fractures, which is related to the fact that the cases we included involved older children and adolescents.

Is the proximal humerus fracture better treated conservatively or surgically in children? Whether conservative or surgical treatment is more suitable for treating proximal humeral fractures in older children and adolescents is still a subject of debate. In regard to the treatment of paediatric proximal humeral fractures, the Neer-Horowitz (N-H) classification system is always mentioned. This classification divides children’s proximal humeral fractures into four types: Types I and II (minimal fracture displacement);​ Type III (fracture is displaced up to 2/3 of the width of the humeral shaft); and Type IV (fracture displacement exceeds 2/3 of the width of the humeral shaft). The N-H classification system is an important basis for guiding the treatment of proximal humeral fracture in children.

The results of a recent Scottish study on the conservative treatment of proximal humeral fracture in adolescents are intriguing. This study included 118 adolescents with a median age of 12 years and who had proximal humeral fracture. Most of them were Type I according to the N-H classification, and 3 were Types III and IV. During the follow-up after treatment, 3 patients (6%) had residual shoulder pain, and 4 patients (11%) had differences in the appearance of both upper limbs. One of the three patients with type III and IV fracture displacement completed the follow-up questionnaire. The shoulder joint of this patient was still painful, and the shoulder joint function score was significantly lower than the average score of all patients. The author believed that conservative treatment could achieve good functional results for significantly displaced proximal humeral fractures in adolescents. Unfortunately, their study included too few cases of significantly displaced fractures and was thus unable to provide an adequate reference for the conservative treatment of severely displaced proximal humeral fractures in children and adolescents [13]. Some studies also compared the results of conservative treatment and surgical treatment of more significantly displaced paediatric proximal humeral fracture (N-H types III and IV) and showed that conservative treatment of proximal humeral fractures in older children may be more likely to lead to unsatisfactory clinical results, especially for patients older than 12 years old. For these patients, the probability of poor prognosis will increase 3.81 times for every 1-year increase in age at the time of injury [14].

Therefore, the current popular view is that treatment should be based on the individual characteristics of each child, taking full account of the patient’s age, degree of fracture displacement, bone plasticity and other factors. Lefèvre et al. [15] divided children with proximal humeral fracture into three age groups: under than 10 years old, 10–13 years old and over 13 years old. They believed that most children under 10 years old can be cured through conservative treatment; for patients over 13 years old, because of their limited fracture plasticity, displaced fracture patients needed surgical treatment. For patients aged 10–13 years, a comprehensive analysis was needed to determine the treatment method. Because the plasticity of bone decreases significantly with age, children over 10 years old may have been unable to rely on bone plasticity to correct 20° angulation [7]. Therefore, it is generally accepted that N-H type III and IV proximal humerus fractures in older children and adolescents should be treated surgically [8, 16]. For this reason, the surgical subjects selected in our study were patients older than 10 years with N-H type III or IV fractures.

The purpose of modern surgical treatment of fractures in children is to achieve satisfactory reduction and fixation of fractures so that they can be healed without residual deformity or dysfunction [17]. The main surgical treatments for paediatric proximal humeral fracture, since the 1980s, include fixation with a Kirschner wire, lag screws and bone plates, and then fixation with elastic intramedullary nails was gradually introduced as a surgical treatment for paediatric proximal humeral fracture [18]. After fracture reduction, multiple Kirschner wire fixation is the most commonly used surgical method for paediatric proximal humeral fracture. Kirschner wire fixation is a simple surgical procedure designed to avoid secondary damage to the growth plate [19]. As a relatively stable method, elastic intramedullary nail fixation is also a commonly used surgical method for paediatric proximal humeral fracture. Bone plate fixation may provide more stability [8], but the current locking plate system has a potential risk of causing iatrogenic epiphyseal plate injury and is only applicable to some adolescent patients with epiphyseal plate closure. Elastic intramedullary nails, lag screws and locking plates need to be removed in a second operation. At present, the application of external fixators in the operation of proximal humeral fracture in children and adolescents is novel, and there are few related studies. In 2013, Lollino et al. used four 2.5 mm Kirschner wires to fix the fracture in two adolescent patients for the first time and then connected the pin tail to a connecting rod to form an external fixation frame to treat Salter-Harris Type II proximal humeral epiphysis injury [20]. The strength of the external fixator was better than that of the Kirschner wire in mechanical analysis studies [21].

Few studies have compared the efficacy of different treatment methods in children with proximal humeral fracture, and only one study in the literature compared the efficacy of Kirschner wire fixation and external fixation. Their results showed that the operation time of external fixation was shorter, and the open reduction rate was lower than that of Kirschner wire surgery. In external fixation, two Schanz screws were placed horizontally at the far and near ends, and two Schanz screws were placed at the far end along the humeral shaft accordingly [22]. The external fixation selected in our study included two Schanz screws and anti-rotating Kirschner wires. The advantages of this surgery are that there is no need for prolonged postoperative immobilization of the affected limb, and functional exercise can be performed in the early postoperative period. The surgical technique is also minimally invasive, safe and simple, and the learning curve is short. The function scores of the shoulder joint in the external fixation group were significantly better than those in the Kirschner wire group in the early postoperative period. However, 9 months after the operation, there was no significant difference in the shoulder joint score between the two groups. With the increase in postoperative rehabilitation time, both groups of patients showed satisfactory shoulder joint function.

**Fig. 2:**
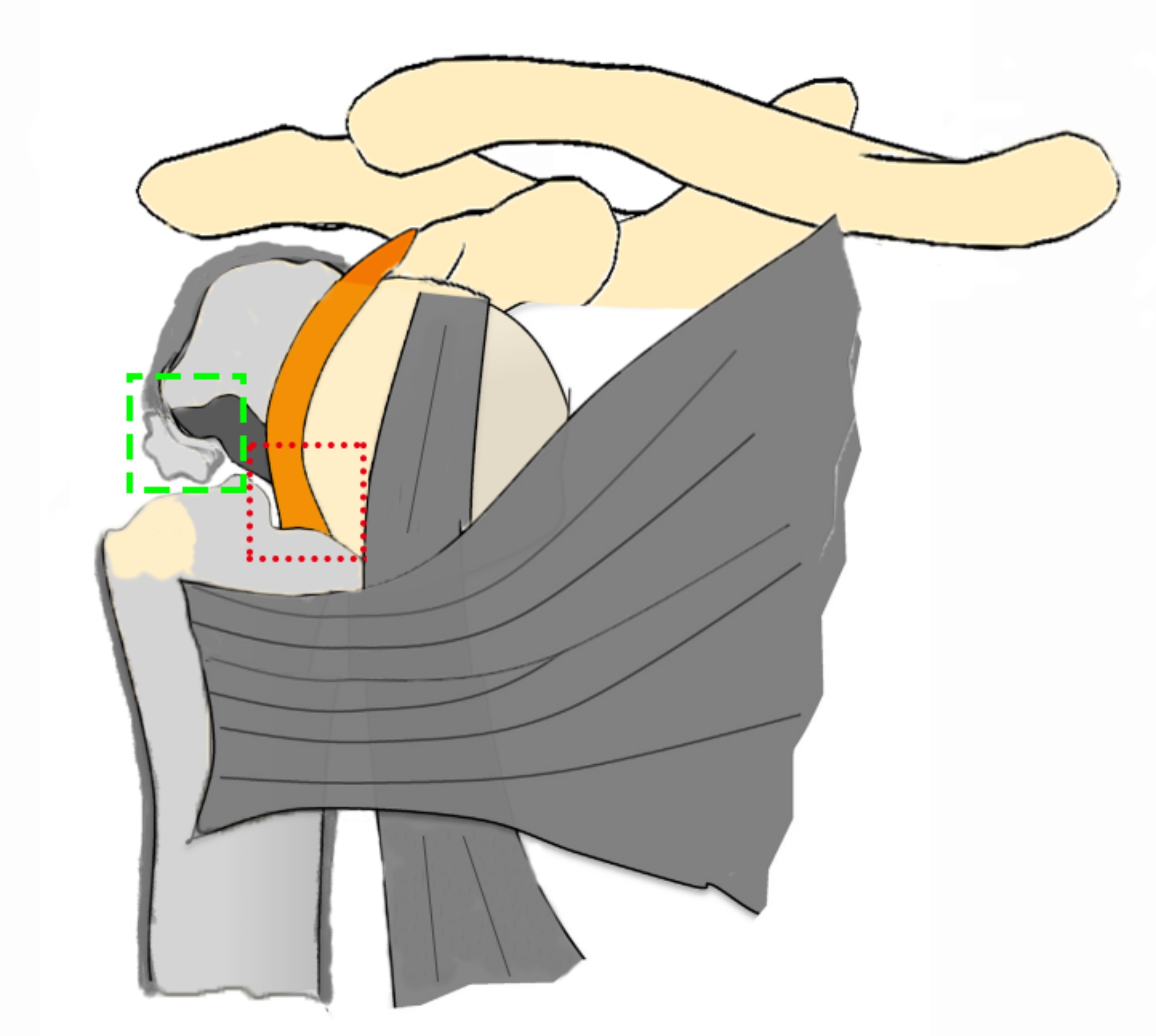
Entrapment of the periosteum and biceps tendon in proximal humeral fracture. (The green area indicates the periosteum; the red area indicates the biceps tendon)

Closed reduction is the first choice for treating proximal humeral fracture, but in some cases, open reduction is needed due to the failure of closed reduction. Research has shown that the open surgery rate of proximal humeral fracture in children is approximately 10% [8]. The periosteum and biceps tendon are the most common tissues that hinder closed reduction during surgery [23] (Fig. 2). In our study, the periosteum was the main tissue that hindered closed reduction. During the operation, it is usually necessary to partially incise the periosteum embedded in the broken end of the fracture to reduce the fracture. Studies have shown that for adolescents with severe displacement of proximal humeral fractures, if closed reduction fails, open reduction can also achieve satisfactory results [23].

The main complication after Kirschner wire fixation and external fixation is pin tract infection. Because the muscles around the shoulder joint are rich, the diameter of the Schanz screw is larger than that of the Kirschner wire. The risk of pin tract infection may be higher in external fixation than in simple Kirschner wire surgery. In our study, the incidence of pin tract infection in the external fixation group was also higher than that in the Kirschner wire group. Fortunately, pin tract infection after surgical fixation of proximal humeral fracture can usually be cured within 2 weeks by oral or intravenous antibiotics [24], and other deep tissue infections rarely occur.

The other possible complications after surgical treatment of paediatric proximal humeral fracture include fixator displacement, secondary fracture displacement, vascular and nerve injury, fracture nonunion, joint stiffness and osteonecrosis. The incidence of these complications is generally low [8] but still needs attention. The possibility of secondary displacement of the Kirschner wire is higher than that of external fixators. In this study, two patients in the Kirschner wire group experienced displacement of the fixators. Because of the small displacement of the Kirschner wires, there was no displacement at the fracture end. Placement of the Kirschner wire and Schanz screw from the outside of the shoulder joint increases the risk of axillary nerve injury. In children and adolescents, the posterior branch of the axillary nerve is located 3.2 to 7.3 cm from the tip of the acromion. Therefore, the pins should be kept away from this area to avoid causing an axillary nerve injury [25]. Anatomical studies have shown that the diameter of the humeral head of adolescents is 41.3 mm and the neck shaft angle is 36°. The two lateral Kirschner wires were ideally positioned 4.4 and 8.0 cm from the proximal end of the humerus head and 21.2° from the long axis of the humerus on the coronal plane [4]. Surgeons should also attempt to avoid damaging the posterior humeral circumflex artery. If allowed, moderate external rotation of the upper arm will reduce the risk of injury to the posterior humeral circumflex artery [26]. Note that the fixed pin should not penetrate the articular cartilage surface of the humeral head or protrude into the joint.

In cases of epiphyseal injury in proximal humeral fractures, shortening of the humerus may also occur, but this shortening is usually less than 2 cm and is well tolerated [15]. Therefore, we did not record or compare humeral shortening in the two treatment groups.

Notably, 40% of all pathological fractures occur in the proximal humerus. If the fracture is caused by low-energy injury or the patient has shoulder pain before the fracture occurs, the possibility of pathological fracture should be considered. Compared with benign bone lesions such as bone cysts, the possibility of osteosarcoma should be considered. Osteosarcoma is the most common primary bone tumour in children and adolescents, with an annual incidence of 5.6 cases per million in children under 15 years of age. The proximal humerus is the common location of osteosarcoma [27, 28]. ​In cases of missed diagnosis and misdiagnosis, the consequences can be catastrophic (Fig. S3-7). Surgeons should remain aware of this possibility during the diagnosis and treatment. Suspected cases should be fully examined, and the operation should be carefully performed.

This study is limited by the nature of its retrospective analysis, and there may be selectivity bias and bias between different operators. Because the incidence of proximal humeral fracture is low, the number of patients in each group is small, and our follow-up time is relatively short, we may not be able to make a more accurate determination of the long-term impact of the two surgical methods on the prognosis of paediatric proximal humeral fracture. However, based on the current results of this study, both Kirschner wire fixation and external fixation for displaced proximal humeral fractures in older children and adolescents can achieve good results.

## Summary

Both Kirschner wire fixation and external fixation surgery for N-H III and IV proximal humeral fractures in older children and adolescents can achieve good results. The learning curve of the two surgical techniques is short, and the operation is safe, simple and minimally invasive. No secondary surgery was needed to remove the internal fixator. The risk of pin tract infection after Kirschner wire surgery is low, but additional plaster immobilization is needed after surgery. After external fixation, the affected limb can be moved early so that the patient can return to their preinjury living status earlier. External fixation is a surgical option for the treatment of displaced proximal humeral fractures in older children and adolescents.

### Electronic supplementary material

Below is the link to the electronic supplementary material.

Supplementary Material 1

Supplementary Material 2

## Data Availability

All data generated or analyzed during this study are included in this manuscript.
