# Supplementary material for: Kirschner wire versus external fixation in the treatment of proximal humeral fractures in older children and adolescents: a comparative study
Source: BMC Musculoskelet Disord. 2023 Nov 18;24:899. doi: 10.1186/s12891-023-07037-x (PMC10656960; doi:10.1186/s12891-023-07037-x)
Supplement: Supplementary file 1 — Supplementary Material 1 [file 12891_2023_7037_MOESM1_ESM.pdf]

Supplementary materials

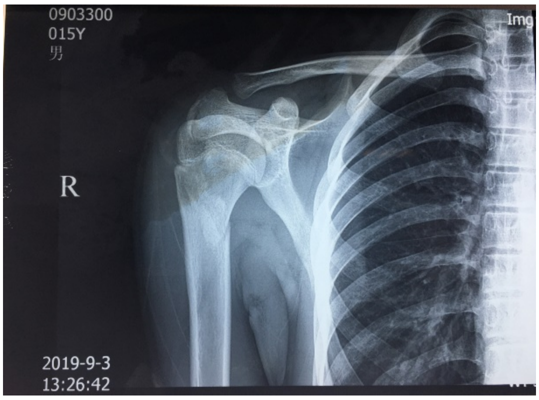

Fig S1. A Preoperative X-ray of a 15-year-old male patient was misdiagnosed as of proximal humerus.

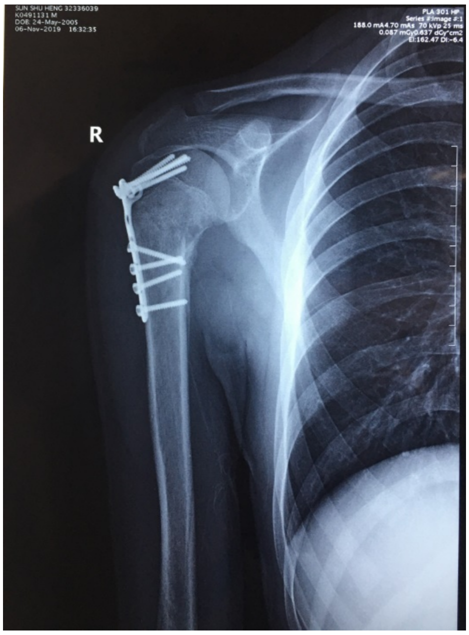

Fig S2. A Postoperative anteroposterior X-ray of the 15-year-old male patient with internal plate fixation for fractures of proximal humerus

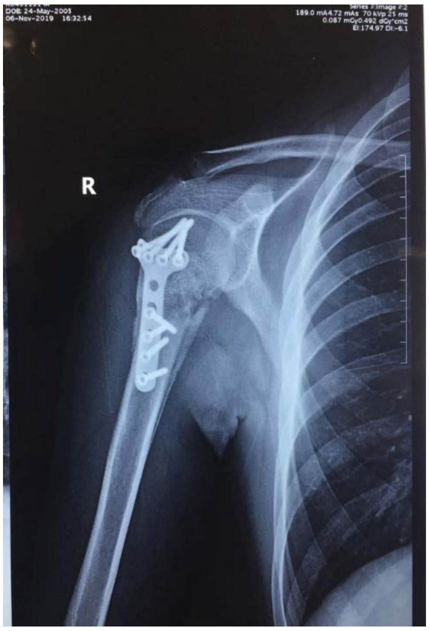

Fig S3. A Postoperative lateral X-ray of the 15-year-old male patient with internal plate fixation for fractures of proximal humerus

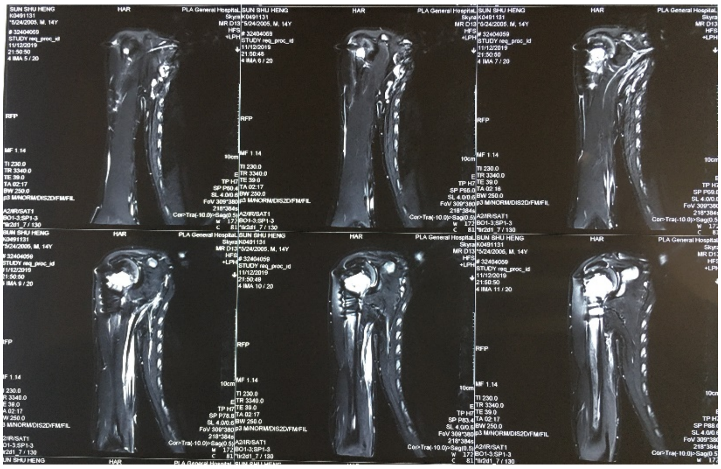

Fig S4. A Postoperative MRI of the 15-year-old male patient diagnosed pathological fracture of osteosarcoma of proximal humerus.

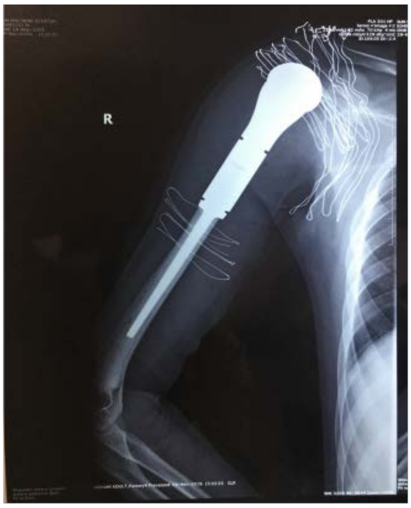

Fig S5. A Postoperative anteroposterior X-ray of the 15-year-old male patient underwent artificial shoulder joint replacement.
