# Supplementary material for: Kirschner wire versus external fixation in the treatment of proximal humeral fractures in older children and adolescents: a comparative study
Source: BMC Musculoskelet Disord. 2023 Nov 18;24:899. doi: 10.1186/s12891-023-07037-x (PMC10656960; doi:10.1186/s12891-023-07037-x)
Supplement: Supplementary file 2 — Supplementary Material 2 [file 12891_2023_7037_MOESM2_ESM.pdf]

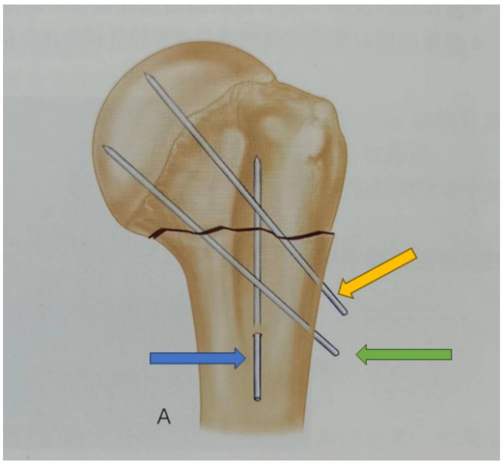

Figure S1. Schematic diagram of Kirschner wires fixation. Yellow and green arrows point to the other 1-2 wires inserted close to the lateral side and fixed from the outside-in direction.

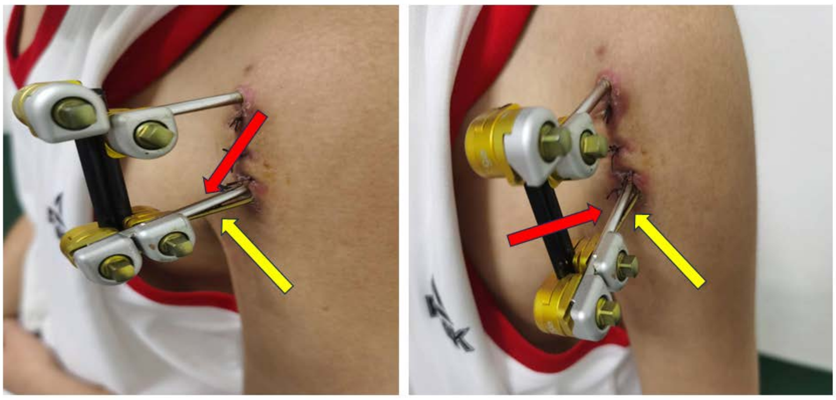

Figure S2. Combined fixation with anti-rotation Kirschner wires and external fixation. The yellow and red arrows in the figure refer to the two anti-rotating Kirschner wires fixed to the external fixation.
